# Reconstructive endovascular treatment of vertebral artery dissecting aneurysms with the Low-profile Visualized Intraluminal Support (LVIS) device

**DOI:** 10.1371/journal.pone.0180079

**Published:** 2017-06-29

**Authors:** Chuan-Chuan Wang, Yi-Bin Fang, Ping Zhang, Xuan Zhu, Bo Hong, Yi Xu, Jian-Min Liu, Qing-Hai Huang

**Affiliations:** 1 Department of Neurosurgery, Changhai Hospital, Second Military Medical University, Shanghai, China; 2 Changhai Stroke Center, Changhai Hospital, Second Military Medical University, Shanghai, China; Universitatsklinikum Freiburg, GERMANY

## Abstract

**Introduction:**

The Low-profile Visualized Intraluminal Support (LVIS) device is a new generation of self-expanding braided stent recently introduced in China for stent assisted coiling of intracranial aneurysms. The aim of our study is to evaluate the feasibility, safety, and efficacy of the LVIS device in reconstructive treatment of vertebral artery dissecting aneurysms (VADAs).

**Methods:**

We retrospectively reviewed the neurointerventional database of our institution from June 2014 to May 2016. Patients who underwent endovascular treatment of VADAs with LVIS stents were included in this study. Clinical presentation, aneurysmal characteristics, technical feasibility, procedural complications, and angiographic and clinical follow-up results were evaluated.

**Results:**

38 patients with VADAs who underwent treatment with LVIS stent were identified, including 3 ruptured VADAs. All VADAs were successfully treated with reconstructive techniques including the stent-assisted coiling (n = 34) and stenting only (n = 4). Post-procedural complications developed in 3 patients (7.9%) including two small brainstem infarctions and one delayed thromboembolic event. Complications resulted in one case of minor permanent morbidity (2.6%). There was no procedure-related mortality. The follow-up angiogram was available in 30 patients at an average of 8.3 months (range, 2 to 30 months), which revealed complete occlusion in 23 patients (76.7%), residual neck in five patients (16.7%), and residual sac in two patients (6.7%). The follow-up of 25 aneurysms with incomplete immediate occlusion revealed 22 aneurysms (88%) with improvement in the Raymond class. One aneurysm (3.3%) showed recanalization and required retreatment. Clinical followed-up at 5–28 months (mean 14.1 months) was achieved in 36 patients because two patients died of pancreatic cancer and basal ganglia hemorrhage, respectively. No new neurologic deterioration or aneurysm (re)bleeding was observed.

**Conclusions:**

Our preliminary experience with reconstruction of VADAs with the LVIS device demonstrates that this treatment approach is feasible with good short-term angiographic and clinical outcomes. Long-term and larger cohort studies are necessary to determine long-term outcomes of this therapy.

## Introduction

Intracranial vertebral artery dissecting aneurysms (VADA) has been recognized as a leading cause of subarachnoid hemorrhage (SAH) and ischemic stroke of the posterior circulation.[1] Endovascular treatment has emerged as the treatment of choice due to perceived lower rates of treatment-related morbidity as well as their efficacy. There are several endovascular approaches including reconstructive techniques using stents alone or in conjunction with coils, and deconstructive techniques with proximal occlusion or internal trapping.[2,3] Compared with deconstructive techniques, reconstructive approaches were shown to be better in maintaining the integrity of the dominant vertebral artery or major branch (PICA or anterior spinal artery) involvement without adequate collateral flow.[4] The rates of long-term occlusion, recurrence, and perioperative mortality are similar between deconstructive and reconstructive techniques in the treatment of VADAs.[5–7] However, aneurysm recanalization secondary to coil compaction and growth of the aneurysm sac remains a major shortcoming of reconstructive treatment.

Endovascular flow diversion (FD), with the ability to reconstruct the parent artery, has been suggested as an important addition to the endovascular treatment for the intracranial arterial dissection. Several articles have reported on the safety and efficacy of the Pipeline embolization device (PED; ev3-Covidien, Irvine, California, USA) for VADAs which showed promising results.[8–11] However, there has been considerable pessimism with respect to the use of PED for posterior circulation aneurysms because of its possible delayed complications, in particular perforator territory infarcts and delayed ruptures.[12–14] Additionally, the China’s Food and Drug Administration (CFDA) sets the indication of PED to treat adults with large or giant wide-necked intracranial aneurysms in the internal carotid artery from the petrous to the superior hypophyseal segments and only a few institutions have been authorized to use the PED in China.[15]

The low profile visualized intraluminal support (LVIS) stent (MicroVention Terumo, Tustin, California, USA), a new device offering an option between conventional neurovascular stents and flow diverters, was designed to improve the long term efficacy of endovascular treatment while avoiding impact on side branches. Its small cell structure (<0.9 mm) and high metal coverage (23% on average) were found to provide great protection across the aneurysm neck and to improve flow diversion over other currently available coil assist stents. However, its safety and efficacy in reconstructive treatment of VADAs have not yet been detailed. We aimed to report the preliminary experience of the LVIS stent in treating VADAs.

## Materials and methods

This retrospective study was approved by our hospital institutional review board; informed consent was aquired prior to study participation.

### Patients and aneurysms

We retrospectively reviewed all patients with VADAs who had been treated with reconstructive technique using the LVIS stent at our institution between June 2014 and May 2016. Three neurointerventionists (J. M. L., Q. H. H., and Y. B. F.) assessed the surgery reports, medical charts, and radiologic images of the patients. Patients’ demographics and presenting symptoms, vascular risk factors, aneurysm characteristics, technical and clinical complications, and follow-up angiography data were evaluated.

**Fig 1:**
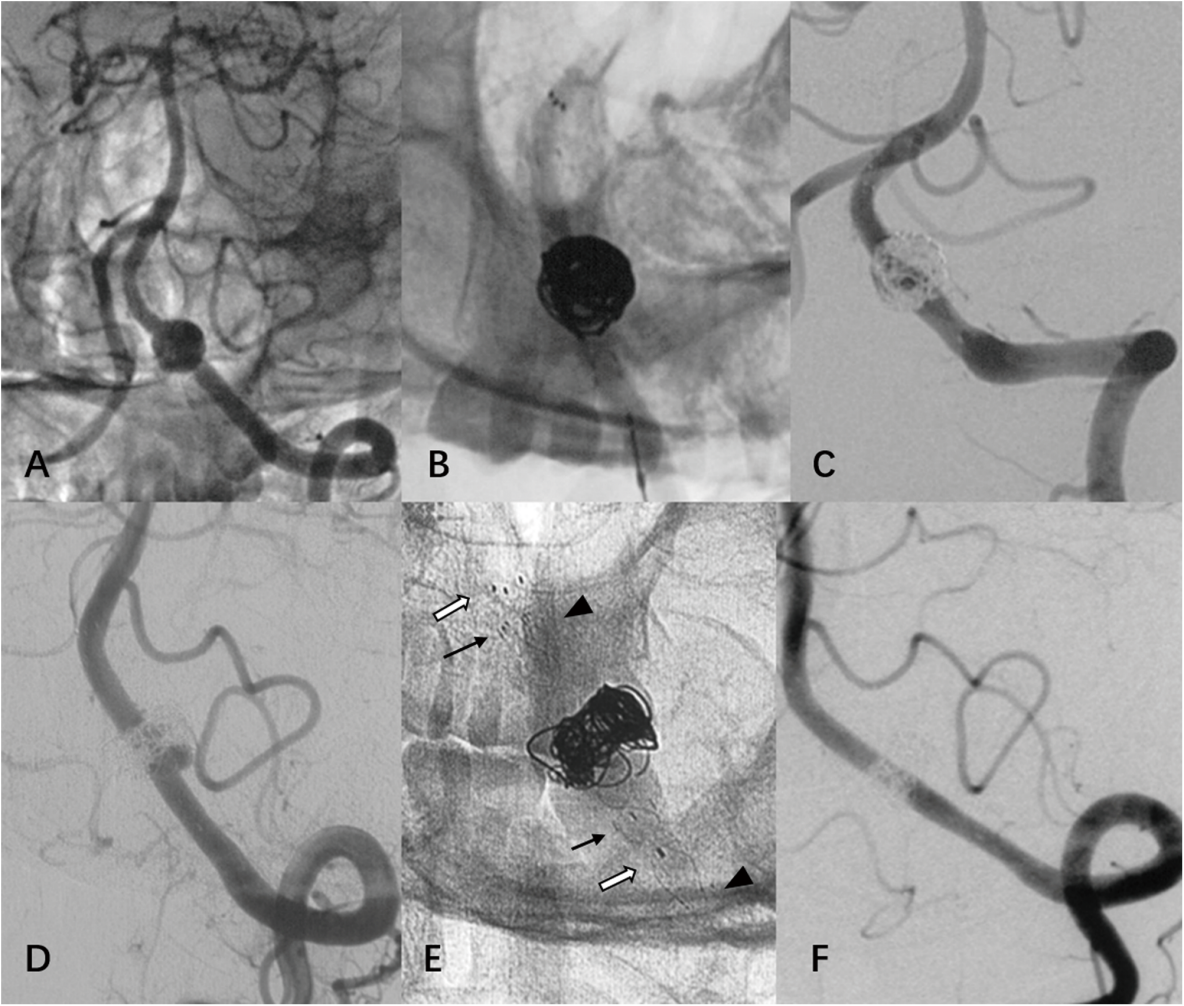
(case 15) A ruptured VADA was reconstructed with one Enterprise (black arrow) and one Solitaire (white arrow) stent assisted coiling, and immediate total obliteration was achieved at a local hospital (A, B, C). After 7 months, a recurrence was observed at the proximal region of VADA and was effectively retreated with an additional LVIS stent (arrowhead) without coiling at our hospital (D, E). A 5-month follow-up angiogram showed normalization (F).

A total of 38 patients with 38 VADAs were identified. The patients consisted of 25 men and 13 women with a mean age of 53.7 years (range 31–73 years). Patient risk factors included hypertension (47.4%), dyslipidemia (34.2%), diabetes (15.8%), and smoking (13.2%). Three patients presented with acute SAH (Hunt-Hess grades: I, III, and V respectively). Two aneurysms had been previously treated using closed-cell stent assisted coiling, but recanalized, and were thus re-treated with LVIS stent (Fig 1). Aneurysms were incidentally found in five cases, whereas the other 28 patients presented with neurological symptoms such as headache in the occipital region or nuchal pain, vertigo, ataxia, syncope, hemiparesis, hemiplegia, or visual changes.

Dominancy of the involved vertebral artery was as follows: dominant in 13 patients, non-dominant in 1 patient, and even in 24 patients. Anatomical findings were classified into four types based on their relationship to the PICA: PICA proximal type in 4 patients, PICA involved in 21 patients, PICA distal in 9 patients, and absent PICA in 4 patients. The mean angiographic length and diameter of the aneurysms were 10.2 mm (range, 2.8–20.6 mm) and 6.0 mm (range, 2.4–10.6 mm) respectively.

### Procedural details

For patients with acutely ruptured aneurysms, a loading dose of clopidogrel and aspirin (300 mg each) was administered orally by gastrointestinal tube or per rectum two hours before stenting. Patients with unruptured VADAs were treated with 75 mg of clopidogrel and 100 mg of aspirin at least 3 days before the endovascular procedure. Heparin was intravenously infused after the femoral sheath was placed, with the goal of achieving an activated clotting time of 2–2.5 times that of baseline during the procedure. Heparinization was discontinued at the end of the procedure. After the procedure, patients were maintained on dual antiplatelet therapy for at least six weeks and after that on aspirin only indefinitely.

All procedures were performed under general anesthesia using biplane angiographic equipment. A 6F guiding catheter or sheath was placed in the distal V2 segment of the vertebral artery. Reconstructive treatment included the stent-assisted coiling or endovascular stenting only. The stent-assisted coiling strategy in our series was the modified stent-assisted semi-jailing with either a single stent or overlapping stents. The stent was semi-deployed to cover the aneurysmal neck after introducing part of the framing coil and additional coils were placed loosely and circumferentially at the aneurysmal dilation through the ‘jailed’ microcatheter. We focused on the most dilated portion of the aneurysm in order to increase the packing density. Stenting only was indicated for aneurysms with important side branches involved and (or) no obvious aneurysmal dilation. Stent sizes were chosen on the basis of the largest diameter of the parent artery and the length of the aneurysm. Our aim of VADA treatment is the complete embolization or obvious contrast stasis. If it is not achieved with a single stent assisted coiling, an additional stent should be manipulated.

Technical success was defined by correct placement of the stent and successful positioning of the coils into the body of the aneurysm without compromising the parent or perforator vessels.[16]

### Clinical assessment, angiographic and clinical follow-up

Clinical outcome was assessed using the modified Rankin Scale (mRS) score at discharge and the last clinical follow-up. Immediate post-procedural angiograms were obtained to evaluate aneurysm occlusion according to the Raymond classification.[17] The first angiographic follow-up was generally performed at three months by using MR angiography (MRA) and the second follow-up performed at six months by using DSA. In-stent stenosis, recanalization, need for retreatment, and patency of jailed perforator arteries were evaluated on follow-up angiogram. Angiographic recanalization was diagnosed when the contrast medium within the lesions substantially increased in comparison with a control angiogram taken immediately after treatment.

## Results

Clinical, demographic, and angiographic data of all patient are detailed in Table 1.

**Table 1:** Clinical data of all the patients.

Pt #	Gender, Age (yrs)	Presentation	H-H grade	Site of dissection	VA dominance	Aneurysm		Strategy	Stent Name, Uncontrained Diameter (mm), Model (size)	Initial Raymond grade	Procedural complication	Angiographic FU		
						Length	Diameter					Time	Results	PICA
1	M, 42	Headache and dizziness	0	PI	E	13.6	6.9	SAC	2 LVIS* 4.5, 213041-CAS, 213015-CAS	3	No	17	1	Patent
2	M, 35	Ischemic infarction	0	PD	D	11.7	6.6	SAC	LVIS* 4.5, 213025-CAS	3	No	30	3, Regrowth	Patent
3	M, 58	Headache	0	PD	E	13.0	11.0	SAC	LVIS 4.5, 213025-CAS, EP 4.5×37	3	No	7	1	Patent
4	M, 60	Ischemic infarction	0	PI	D	20.0	9.3	SAC	LVIS 5.5, 214035-CAS, EP 4.5×37	3	No	12	1	Patent
5	F, 48	Ischemic infarction	0	Absent	E	7.5	6.0	SAC	2 LVIS 3.5, 212525-CAS*2	1	No	7	1	NA
6	F, 52	Neck pain	0	PI	E	4.3	3.3	SAC	2 LVIS 3.5, 212525-CAS*2	3	No	10	1	Patent
7	F, 44	Ischemic infarction	0	PI	D	10.6	5.2	SAC	LVIS 3.5, 212525-CAS	1	No	8	1	Patent
8	M, 59	Mass effect	0	PI	E	13.8	10.1	SAC	2 LVIS 4.5 213025-CAS*2	3	No	No FU		
9	M, 73	Dizziness	0	PI	D	20.6	5.3	SO	LVIS 3.5, 212525-CAS, EP 4.5×37	3	Delayed PA occlusion	2	1	Occluded
10	M, 52	Mass effect	0	PP	D	4.8	3.2	SAC	LVIS 3.5, 212517-CAS	3	No	11	1	Patent
11	M, 47	Dizziness	0	PI	E	15.5	8.2	SAC	2 LVIS 4.5&5.5, 213041-CAS, 214035-CAS	3	No	9	3	Patent
12	M, 46	Asymptomatic	0	PD	E	9.4	5.2	SAC	2 LVIS 4.5, 213041-CAS, 213025-CAS	3	Pontine infarction	No FU		
13	M, 61	Asymptomatic	0	PI	E	4.0	2.8	SAC	LVIS 4.5, 213025-CAS	1	No	13	1	Patent
14	M, 31	Recanalized	0	PI	ND	8.0	4.4	SAC	2 LVIS 4.5, 213025-CAS*2	3	No	5	1	Patent
15	M, 54	Recanalized	0	Absent	D	8.1	8.9	SAC	LVIS 4.5, 213025-CAS	3	No	5	1	NA
16	F, 71	Mass effect	0	PP	E	14.4	10.4	SAC	LVIS 4.5, 213041-CAS	3	No	Deceased		
17	F, 43	SAH	2	PD	E	9.4	5.9	SAC	LVIS 3.5, 212525-CAS	3	No	7	1	Patent
18	M, 59	Dizziness	0	PI	E	6.6	3.2	SAC	2 LVIS 3.5&4.5, 212525-CAS 213041-CAS	2	No	7	1	Patent
19	M, 58	Mass effect	0	PI	E	12.7	5.4	SAC	2 LVIS 4.5, 213041-CAS, 213025-CAS	3	No	7	2	Patent
20	M, 43	SAH	5	PI	D	5.7	3.5	SAC	2 LVIS 3.5, 212525-CAS*2	2	No	7	1	Patent
21	F, 60	SAH	3	PD	E	6.6	4.1	SAC	2 LVIS 3.5, 212525-CAS, 212517-CAS	1	No	6	1	Patent
22	F, 69	Neck pain	0	PI	D	7.6	4.7	SAC	2 LVIS 4.5, 213025-CAS, 213015-CAS	3	Pontine infarction	8	2	Patent
23	F, 46	Headache	0	PP	E	3.9	2.4	SAC	LVIS 3.5, 212525-CAS	2	No	No FU		
24	M, 63	Sycope	0	PI	D	6.2	3.4	SAC	2 LVIS 3.5, 212517-CAS*2	1	No	8	1	Patent
25	M, 44	Ischemic infarction	0	PI	E	10.5	8.7	SAC	LVIS 4.5, 213025-CAS	3	No	6	1	Patent
26	M, 43	Headache	0	PI	E	16.0	7.6	SAC	2 LVIS 4.5, 213041-CAS, 213025-CAS, EP 4.5×37	3	No	6	2	Patent
27	M, 48	Headache	0	PD	E	4.4	2.9	SAC	2 LVIS 3.5, 212517-CAS*2	2	No	7	1	Patent
28	F, 70	Asymptomatic	0	PD	D	16.5	10.6	SAC	LVIS 4.5, 213025-CAS, EP 4.5×37	2	No	9	2	Patent
29	M, 63	Ischemic infarction	0	PD	E	15.8	10.2	SAC	2 LVIS 4.5, 213041-CAS*2	3	No	Deceased		
30	F, 52	Headache	0	PI	E	9.6	4.9	SAC	2 LVIS 3.5, 212525-CAS*2	3	No	No FU		
31	F, 43	Headache	0	PI	E	2.8	2.5	SAC	LVIS 3.5, 212517-CAS	3	No	6	1	Patent
32	M, 55	Ischemic infarction	0	PI	D	11.4	6.9	SO	2 LVIS 3.5&4.5, 214035-CAS, 213041-CAS	3	No	6	1	Patent
33	F, 67	Vertigo	0	PP	D	4.8	3.8	SAC	LVIS 3.5, 212525-CAS	3	No	6	1	Patent
34	M, 66	Syncope	0	PI	D	16.1	7.5	SAC	LVIS 4.5, 213041-CAS	3	No	7	1	Patent
35	M, 54	Ischemic infarction	0	Absent	E	8.2	4.4	SAC	2 LVIS 4.5, 213025-CAS*2	3	No	5	2	NA
36	M, 35	Asymptomatic	0	PD	E	11.1	6.8	SO	2 LVIS 4.5, 213041-CAS, 213025-CAS	3	No	6	1	Patent
37	F, 55	SAH	1	PI	E	6.2	4.7	SAC	LVIS 3.5, 212525-CAS	3	No	No FU		
38	M, 54	Asymptomatic	0	Absent	E	15.3	6.9	SO	2 LVIS 4.5, 213041-CAS*2	3	No	No FU		

Absent, no posterior inferior cerebellar artery (PICA); D, dominant; E, even; EP, Enterprise stent; FU, follow up; Lt, left; LV, LVIS blue stent; NA, not applicable; ND, non-dominant; PA, parent artery; PD, PICA distal; PI, PICA involved; PO, proximal occlusion; PP, PICA proximal; Pt, patient; Rt, right; SAC, stent-assisted coiling; SAH, subarachnoid hemorrhage; SO, stenting only; UE, upper extremity; VA, vertebral artery.

* LVIS pre-blue version stent

### Technical success and immediate angiographic results

**Fig 2:**
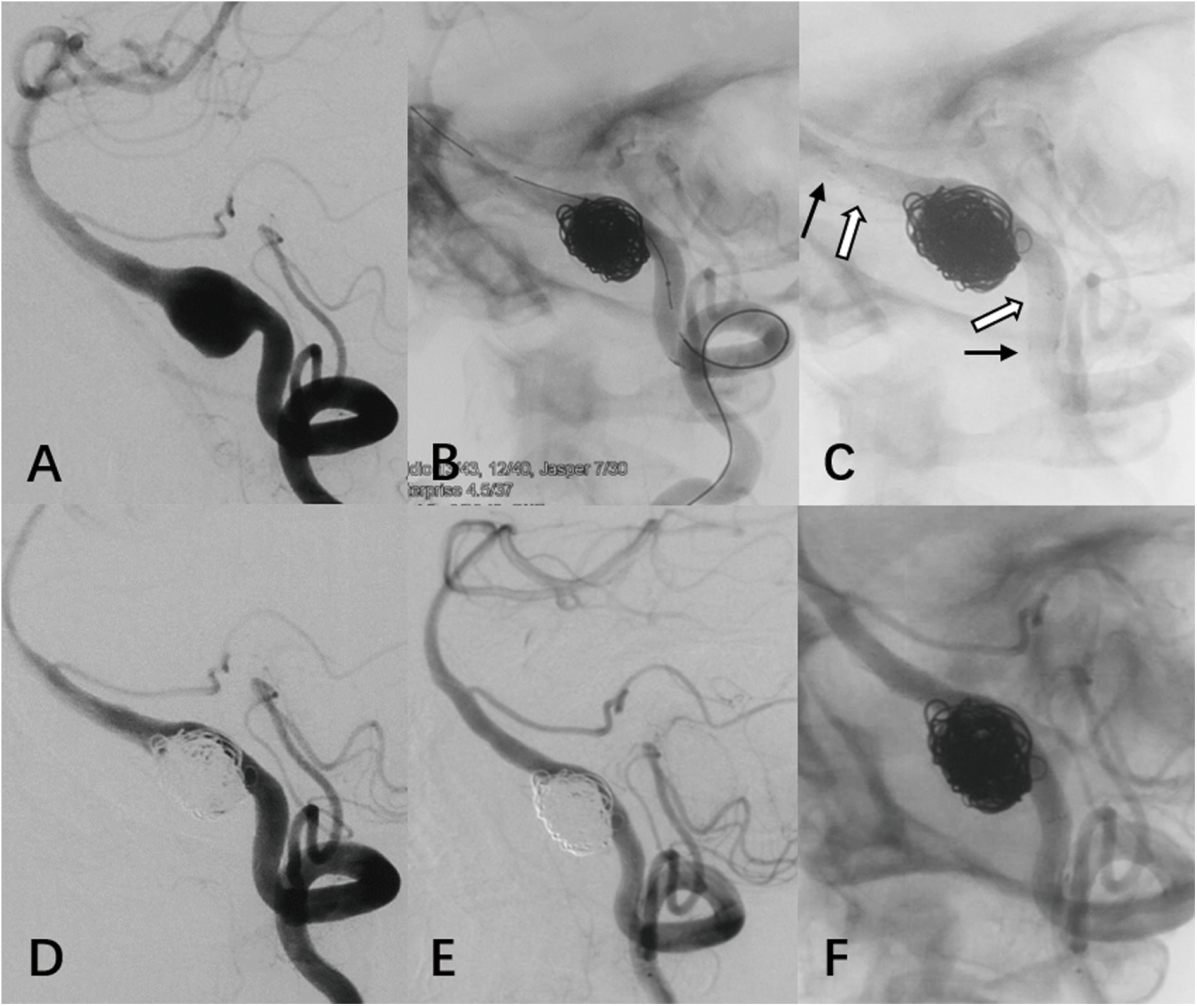
(case 3) Left VA angiography detected a VADA with a long configuration and tortuous parent artery (A). Modified stent-assisted semi-jailing was first manipulated with a 4.5×37 mm Enterprise stent (black arrow). An additional LVIS stent (white arrow) was then deployed (B, C). Immediate post treatment angiography (D) and 7-month follow-up (E, F) demonstrated progressive vessel remodeling and aneurysm occlusion.

All VADAs were successfully treated with reconstructive techniques including the stent-assisted coiling (n = 34) and stenting only (n = 4). Seventeen cases were reconstructed with a single LVIS stent including four patients using another Enterprise stent simultaneously (Fig 2). Double LVIS stents were used in 21 patients including one patient with Enterprise stent used simultaneously.

On post-procedural angiogram, 5 of 38 (13.2%) aneurysms treated with stent-assisted coiling were occluded without remnant. Of the remnant 33 aneurysms with subtotal occlusion after treatment, 5 had residual neck filling (Raymond Class 2), whereas the other 28 had filling of a residual dome (Raymond Class 3). In the four patients treated by stenting alone, contrast residual time within the aneurysm was increased moderately after the stent placement.

### Procedural complications and clinical outcome

We did not experience any intra-procedural complications. Post-procedural complications developed in 3 patients (7.9%) including two small pontine infarctions and one delayed thromboembolic event. Complications resulted in one case of minor permanent morbidity (2.6%). There was no mortality.

**Fig 3:**
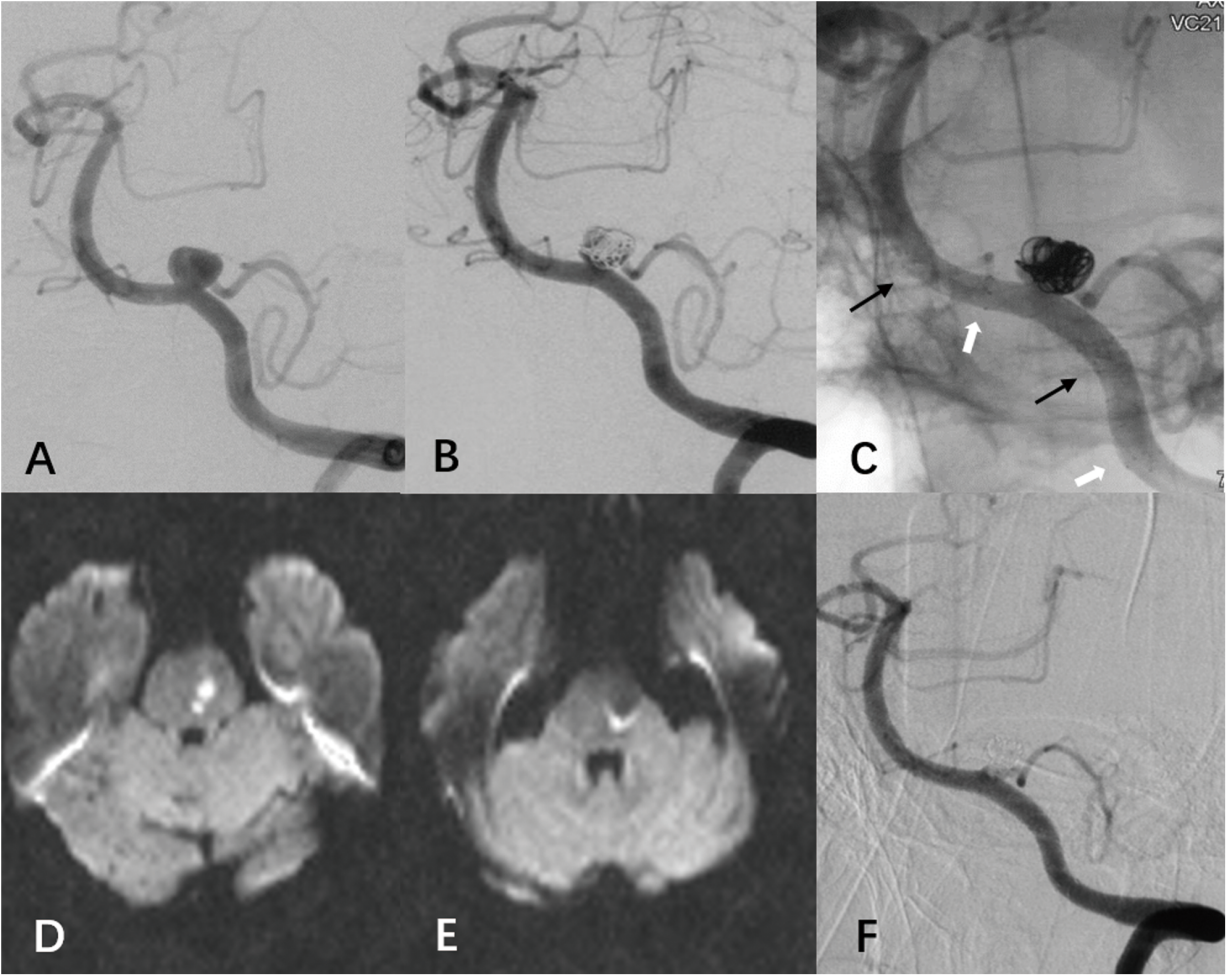
(case 22) The left vertebral angiography at admission showed a PICA-involved VADA, with the PICA arising from the lesion (A). We performed double overlapping LVIS stents (black arrow and white arrow) and loose coiling packing. The immediate angiographic result was partial occlusion of the VADA with the patency of involved PICA (B, C). This patient suffered from mild vertigo and hoarseness after treatment and MR imaging revealed two tiny punctate foci of diffusion restriction in the lower pon(D, E). The angiographic follow-up at 8 months showed progressive aneurysm occlusion without compromise of PICA (F).

Two patients developed ischemic events immediately after procedure. After placement of two overlapping LVIS stents, one patient developed hemiplegia and diffusion-weighted MRI showed a minor infarction in the upper medulla and lower pons (Case 12). An emergent contrast-enhanced MRA at 1.5T was performed and confirmed the patency of the parent artery. We assumed the infarction was associated with a small perforator occlusion. This patient regained his independence at discharge (mRS = 2) and recovered totally at his last follow-up (mRS = 0). Another patient suffered from mild vertigo and hoarseness after treatment of a left VADA by using double LVIS stents assisted coiling (Fig 3, Case 22). MR imaging revealed two tiny punctate foci of diffusion restriction in the lower pons. This patient did not develop any neurologic deficits.

One delayed thromboembolic event developed in an additional patient who experienced hemiplegia and hypoesthesia of face and hemi-lateral body two months after double stents deployment (one LVIS stent and one Enterprise stent) (Case 9), one week after clopidogrel discontued. MR with diffusion demonstrated multiple infarction at the cerebella and parietal lobe. This patient’s emergent DSA revealed complete in-stent thrombosis and total occlusion of vertebral artery V4 segment. Intra-aterial thrombolysis by using IIb-IIIa inhabitor (tirofiban) perfusion via microcatheter was performed but failed and the microcatheter also failed to penetrate the occlusion to the distal end for thrombectomy. Platelet inhibition was tested by evaluating the performance of thromboelastography (TEG Hemostasis System, Haemoscope Corporation, Niles, IL, USA) and a poor response (platelet aggregation inhibition <30%) to aspirin was found. Clopidogrel was resumed and MRA at six-month follow-up showed the occlusion as before; his final follow-up mRS score was 1.

One SAH patient with initial H-H Grade 5 was achieved a mRS score of 4 at discharge. The mRS score in 7 months clinical FU improved to 1. In summary, 37 patients were independent with a mRS score of 0–2 at discharge while only one patient was dependent (case 21, mRS = 3).

### Angiographic and clinical follow-up

Of 36 survived patients (94.7%), a follow-up DSA was available in 30 patients (83.3%) at an average of 8.3 months (range, 2 to 30 months). The final follow-up angiograms revealed a class 1 occlusion in 23 patients (76.7%), class 2 in five patients (16.7%), and class 3 in two patients (6.7%). The follow-up of 25 aneurysms with a partial immediate occlusion (Raymond class 2 or 3) revealed 22 aneurysms (88%) with improvement in the Raymond class (progressive occlusion). One aneurysm (3.3%) showed recanalization and required retreatment. This patient was treated with single LVIS stent assisted coiling and achieved an initial residual neck. The seven-month follow-up angiography revealed minor recurrence and the residual sac showed regrowth at the thirty-month follow-up. The patient’s retreatment was then performed with another LVIS stent. Of the four aneurysms treated with stenting alone, follow-up angiography was obtained in three cases. Two cases demonstrated complete aneurysm occlusion without recanalization during the observation period, whereas the other one suffered delayed in-stent thrombosis as mentioned before.

None of these patients with follow-up angiography demonstrated the evidence of in-stent stenosis. Eighteen dissections with PICA involved underwent angiographic follow-up and none of them showed impaired flow of PICAs except one occlusion because of in-stent thrombosis.

Clinical followed-up at 5–28 months (mean 14.1 months) was achieved in 36 patients because two patients accidentally died during follow-up. One patient died of unexplained intracerebral hemorrhage five months after the procedure. This patient underwent contrast-enhanced MRA (1.5T) follow up one month after the procedure which showed a stable embolization of aneurysm. The hematoma was located at the contralateral basal ganglia and considered irrelevant to the treated aneurysm. However, antiplatelet therapy after stent deployment may increase hemorrhage risk which should not be excluded from its causes. The other patient died of pancreatic carcinoma one and a half years after the aneurysm treatment. Of 36 survived patients, no new neurologic deterioration or aneurysm (re)bleeding was observed.

## Discussion

The LVIS device is a relatively new self-expandable stent designed as an adjunct to coil embolization of wide-necked intracranial aneurysms. The LVIS stent, braided with a single nickel titanium wire that provides approximately 23% surface metal coverage, facilitates improved flow diversion over other currently available coil assist stents. At the same time, reconstruction of the parent artery is achieved with a supportive and flexible device that maintains blood flow through the parent artery and major side branches as well as perforators. To our knowledge, no studies to date have specifically reviewed the evidence for the LVIS stent in reconstructive treatment of VADAs.[18] This study shows that reconstruction of VADAs using LVIS device is feasible with good clinical and anatomical results.

Among various endovascular techniques, reconstructive techniques such as stent placement and stent-assisted coiling are associated with overall lower rate of perioperative morbidity resulting from sacrifice of the parent vessel compared with deconstructive techniques such as parent artery occlusion and trapping.[7,19] However, there are some limitations to be considered with reconstruction of VADAs. Because of the high porosity of previous generations of stents, reconstructive treatment sometimes has the risk of aneurysm recurrence, especially for PICA-involving lesions that often requires the aneurysm sac to be left partially open to ensure adequate PICA flow.[20,21] To reduce the risks, the only way may be the increased potential to cover the aneurysm neck and the PICA orifice by the stent struts and promote neointima formation along the stent struts.[22] Although the stent-in-stent approach may be a promising solution, it was still unknown whether the overlapping conventional stents could indeed provide enough hemodynamic effects, and the increase in procedural risks and costs with placement of multiple devices should also be considered.

The reduced porosity of LVIS device and increased metal coverage area allow improved flow diversion effects compared with other conventional coil assist stents. Wang et al.[23] quantified the difference of fluid diverting effect of the LVIS device compared with the PED and the Enterprise stent using computational fluid dynamics. They found that a single LVIS stent caused more flow reductions than the double-Enterprise stent but less than a PED. Nevertheless, the double-LVIS stent resulted in a better flow diverting effect than a PED. Although in virtual models, the result may provide theoretical basis to flexibly select single or overlapping LVIS stent(s) deployment to avoid the compromise of side branches while ensure the treatment durability. In our series, despite a lower immediate total occlusion rate, progressive occlusion was detected in most of the cases without immediate total occlusion. Only one aneurysm (3.3%) showed recanalization and underwent retreatment. The origin of the PICA was covered in 18 follow-up cases by single or double LVIS stent(s), and there was no evidence of flow compromise of PICAs except one occlusion because of in-stent thrombosis.

In our case series, the LVIS stent was not the only device used during the procedure. Five enterprise stents were simultaneously used with single or double LVIS stent(s) in five patients. As a laser-cut and close-cell design, the Enerprise stent offers several advantages while the braided stent can not provide. On the one hand, the Enterprise stent have greater radial force than the braided LVIS stent and we believe this offers other advantages. If one of the mechanisms of healing is the ‘tacking down’ of the intimal flap, then a stent with greater radial force should have a theoretical advantage. In addition, higher radial force means greater straightening ability of the parent vessel. Changes in the angulation of the parent vessel seem to facilitate aneurysm thrombosis and parent vessel reconstruction.[24] On the other hand, the maximum length of LVIS stent is 30 mm which may be insufficient to cover some lengthy leisions, and its braided design makes it more prone to shorten when deployed. However, the Enterprise stent has a maximum of 37 mm and can maintain its original length which is more suitable for those lengthy aneurysms. Therefore, in some cases with curved vascular geometry and lengthy lesion, an Enterprise stent was first used and then a LVIS stent was deployed to provide better flow diversion effects. The LVIS stent with braided morphology and full length visualization design can provide greater conformability, visibility, and apposition to the vessel wall during the procedure (Fig 2).

Ischemic complications, including perforator infarction and in-stent thrombosis, occurred at both acute stage and delay stage. The total incidence of symptomatic ischemic events in our case series was 7.9% (3/38). One case developed delayed in-stent thrombosis which was related to the poor response to aspirin. Perforator infarction contributed to two ischemic events which may be explained by coverage of the perforator orifice by stent wires because both patients were treated with overlapping LVIS stents. Other potential causes of perforator thrombosis include poor apposition of the stent to the aneurysm wall, migration of disintegrated thrombus formed in the stent, or endothelial injury triggering platelet aggregation. Therefore, the overlapping LVIS stents may increase the flow compromise risks of small perforators. When stent-in-stent approach was manipulated with LVIS stents, the length of overlapping region should be controlled to avoid covering important small side branches.

There were several limitations in our study, including its retrospective design, patient selection bias, the limited number of cases in a single institution, and the short-term follow-up period. The characteristics of flow diverter devices appear to be ideal for the treatment of VADAs, as was also proved in several studies.[8,9,25,26] Actually, PED has been available in China for only 2 years, and the experience of PED in treating posterior circulation aneurysms is limited in our center, even in China. However, we do have published our result of VADAs treated by Chinese flow diverter named Tubridge.[8] In our further study, we will definitely evaluate the efficacy of different treatment strategies in VADAs. Additionally, the stenting strategy was mainly depend on our previous experiences on the reconstructive treatment of VADAs.[21,22] A comparative effectiveness research of different stenting strategies would be further studied in a larger corhort.

## Conclusion

Our preliminary experience with reconstruction of VADAs with the LVIS device demonstrates that this treatment approach is feasible with good short-term angiographic and clinical outcomes. Given the complex technique and increased metal coverage rate, overlapping multiple stents could be related to increased ischemic complications, which should be taken into consideration before using this technique. However, long-term and larger cohort studies are needed to validate these Results.

## Supporting information

S1 File STROBE checklist. (DOCX)
